# Natural Killer Cell-Based Cancer Immunotherapies: From Immune Evasion to Promising Targeted Cellular Therapies

**DOI:** 10.3389/fimmu.2017.00745

**Published:** 2017-07-12

**Authors:** Erhard Hofer, Ulrike Koehl

**Affiliations:** ^1^Department of Vascular Biology, Medical University of Vienna, Vienna, Austria; ^2^Institute of Cellular Therapeutics, IFB-Tx, Hannover Medical School, Hannover, Germany

**Keywords:** immunotherapy, natural killer cells, immune evasion, cell therapy, checkpoint inhibitors, chimeric antigen receptors, bispecific antibodies

## Abstract

Immunotherapies based on natural killer (NK) cells are among the most promising therapies under development for the treatment of so far incurable forms of leukemia and other types of cancer. The importance of NK cells for the control of viral infections and cancer is supported among others by the findings that viruses and tumors use a multitude of mechanisms to subvert and evade the NK cell system. Infections and malignant diseases can further lead to the shaping of NK cell populations with altered reactivity. Counter measures of potential therapeutic impact include the blocking of inhibitory interactions between NK cell receptors and their cellular ligands, the enhancement of activating receptor signals, and the infusion of large numbers of *ex vivo* generated and selected NK cells. Moreover, the specific cross-linking of NK cells to their target cells using chimeric antigen receptors or therapeutic bi-/trispecific antibody reagents is a promising approach. In this context, NK cells stand out by their positive effects and safety demonstrated in most clinical trials so far. Based in part on results of the recent EC-sponsored project “NATURIMMUN” and considering additional published work in the field, we discuss below new developments and future directions that have the potential to further advance and establish NK cell-based therapies at the clinics on a broader scale.

## Introduction

Natural killer (NK) cells have been classically defined as part of the innate immune system providing immediate reactivity against their main targets, virally infected and tumor cells (1). This view has been substantially extended over the recent years based on the findings that NK cells are calibrated to provide self-tolerance, can develop a memory, and play a role in the regulation of the adaptive immune response (2–5). Furthermore, NK cells have turned out to be part of a larger family of innate lymphoid cells (ILCs) that include ILC1–3 (6).

Natural killer reactivity, including cytokine secretion and cytotoxicity, is controlled by a balance of several germ-line encoded inhibitory and activating receptors such as killer immunoglobulin-like receptors (KIRs) and natural cytotoxicity receptors (NCRs) (1, 5, 7, 8). Evidence for the anticancer efficacy of NK cells comes from allogeneic or haploidentical hematopoietic stem cell (HSC) transplantations that have been used in combination with chemotherapy in the treatment of different forms of leukemia (9). This has shown that NK cells formed from the transplant not only are efficient in killing of allogeneic leukemia cells but are also instrumental in reducing the incidence of graft versus host disease due to their killing activity for dendritic cells (10). Taken together with clinical NK infusion trials in leukemia patients, which have shown exciting antitumor activities and generally safety of the procedure (11–13), it appears that NK cells could be the cells of choice in cellular therapies of leukemia not displaying the critical graft versus host activities of T lymphocytes. Although it is currently less clear whether NK cells will be similarly active in solid cancers, this is a further important area of interest.

## Immune Evasion Mechanisms and Shaping of the NK Cell Compartment

Given the importance of NK cells, it is not astonishing that viruses and tumors use a wide array of mechanisms to avoid recognition by NK cells. A paradigm is represented by the Herpes virus family. Many mechanisms such as expression of viral ligands for inhibitory receptors have been described (14). Important is further the downregulation of human stress-induced ligands recognized by the activating NKG2D receptor present on the majority of NK cells. Normally, these stress ligands appear on the cell surface whenever a cell is virally infected or undergoes oncogenic transformation. Whereas internalization and miRNA-mediated downregulation of several stress ligands have been shown previously (15), additional novel mechanisms have been recently identified within the EC-funded project NATURIMMUN. For example, in the case of HHV-6B the expression of stress ligands is suppressed by proteasomal degradation induced by the virus. Consequently, HHV-6B-infected cells can evade immune surveillance by NK cells (16). These various evasion mechanisms of Herpes viruses are reviewed (17) within this research topic (“Tailoring NK Cell Receptor-Ligand Interactions: an Art in Evolution”).

Extending the importance of NKG2D ligands to tumors, Schmiedel et al. have shown within the NATURIMMUN project that the stress ligand ULBP2 can be suppressed by an RNA-binding protein that is frequently overexpressed in tumor cells. By binding of this oncogenic protein to ULBP2 mRNA the stability of the mRNA is reduced and ULBP2 levels on the cell surface are downregulated. In consequence, the tumor cells are protected from NK cell recognition (18). This strongly supports that modulation of stress ligands is an important escape mechanism used by cancer cells to diminish NK cell recognition. Involving a different inhibitory receptor, another unexpected novel evasion mechanism could be shown by the same group for colon cancer. NK cell killing was inhibited by the presence of fecal bacteria in the tumor environment. Bacterial proteins interacted with the inhibitory TIGIT receptor on NK cells leading to the inhibition of NK cell cytotoxicity (19). Inhibition of NK cells can also occur by blocking of NKG2D *via* soluble forms of the stress ligand MICA as shown for neuroblastoma as well as head and neck carcinoma. This tumor escape can be overcome in part by highly activated NK cells with upregulated NKG2D (20, 21).

Viruses and human cancers can further have profound effects on and shape the NK cell compartment. Human cytomegalovirus (HCMV), a herpes family member, can trigger an adaptive NK cell response leading to the expansion of NK cell subsets with specific receptor expression (22–24), e.g., the activating NKG2C receptor. The adaptive NKG2C NK cells have been implicated in improved survival of leukemia patients receiving a HSC transplant from HCMV-positive donors (23, 25). Given the potential higher antitumor reactivity of the NKG2C NK cells, this subset is of therapeutic interest and was investigated within the frame of the NATURIMMUN project. Obtained results support that different adaptive NK cell subsets develop in response to viral infection and this is influenced by the copy number of the NKG2C gene (26).

It has been established that certain forms of leukemia display a defective NK cell compartment (27) rendering these forms priority cases for the exploration of NK cell-based therapies. In regard of acute myeloid leukemia (AML), we investigated within the NATURIMMUN project NK cells in patients receiving a novel maintenance therapy with histamine plus IL-2. In this study, AML patients displayed diminished and partly defective NK cells. The therapy strongly induced the immunomodulatory CD56^bright^CD16^−^ and CD56^bright^CD16^low^ NK cell subtypes and contributed to the restoration of the NK cell compartment (28). This is in line with the described positive effects of the therapy on disease-free survival of AML patients (29, 30). In addition, our cooperation partner S. Huenecke describes in this research topic that during immune reconstitution after HSC transplantation the degree of development of the two CD56^bright^ and the CD56^dim^ NK cell subpopulations can serve as prognostic marker for both graft versus host disease and viral infections (31).

## Modulation of Inhibitory NK Receptor–Ligand Interactions and Novel Ligands of Activating Receptors

Unprecedented rates and durations of clinical responses have been recently achieved in cancer patients by the treatment with antibody reagents that block inhibitory “checkpoint receptors” (32). Whereas these therapies have so far been restricted to the blockade of inhibitory pathways acting on T lymphocytes, the inhibition of NK cells by the interaction of inhibitory NK cell receptors with MHC class I ligands can be regarded as typical checkpoint inhibition. In fact, efforts are currently been undertaken to evaluate blockade of the inhibitory NKG2A/CD94 receptor and of inhibitory KIRs to elicit NK reactivity to cancer cells. The company Innate Pharma has developed first-in-class monoclonal antibodies that target inhibitory NK cell receptors and these are currently in preclinical and clinical evaluation (33).

While the ligands for inhibitory NK cell receptors are well established, ligands bound by important activating receptors are still incompletely identified. This is the case for the activating NKG2C/CD94 receptor, several activating KIRs, and the NCRs. In this regard, a group participating in NATURIMMUN has studied how HCMV stimulates NK cells *via* the activating KIR2DS1 receptor. The ligand was identified as a specific class I molecule, HLA-C2, which in its normal form is recognized by the related inhibitory KIR2DL1 receptor. Possibly, a conformational change in normal HLA-C2 triggered by HCMV was required for KIR2DS1-mediated NK cell activation (34). Other participants in NATURIMMUN have developed assay systems and have work in progress to identify virally induced and potentially tumor ligands for the activating NKG2C receptor (Pupuleku et al., manuscript in preparation for this research topic) and the NCRs The clarification of the molecular nature and mechanism of action of the corresponding activating ligands on virally infected and tumor cells will allow novel pathways of NK cell activation to be triggered.

## Generation of Large-Scale Therapeutic NK Cells and Technology to Target and Cross-Link NK Cells to Cancer Cells

**Figure 1:**
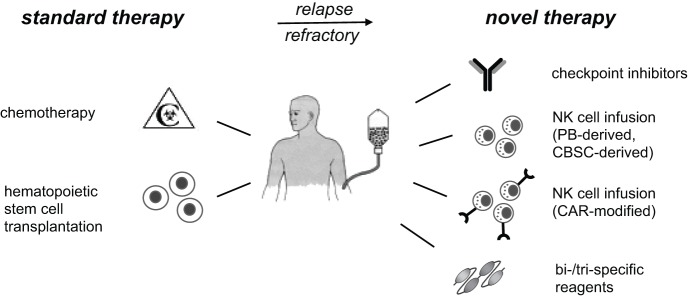
NK cell-based immunotherapies. Standard therapy for high-risk leukemia includes high-dose chemotherapy, followed by hematopoietic stem cell transplantation. Patients, who do not reach remission or suffer from early relapse thereafter, have a poor prognosis and are in urgent medical need for advanced therapies. Current immunotherapeutic developments and phase I/II trials include checkpoint inhibitors for inhibitory NK receptors, infusion of expanded and activated autologous or allogeneic NK cells, and targeting of NK cells to cancer cells. The latter can be done by modification of NK cells with CAR or by application of multispecific reagents to cross-link NK cells with cancer cells. These immunotherapies should reduce relapse rates and constitute promising additional treatment options for high-risk patients. PB, peripheral blood; CBSC, cord blood stem cell; CAR, chimeric antigen receptor; NK, natural killer.

Exploiting and strengthening the NK cell response is a highly promising approach for future successful immunotherapies of cancer. This could be achieved by infusion of *ex vivo* expanded and activated NK cells, by genetic modification of NK cells with chimeric antigen receptors (CAR), by multivalent reagents cross-linking NK cells to cancer cells, or by a combination of these methods (Figure 1).

In regard of *ex vivo* expansion of peripheral donor NK cells several groups have developed corresponding technologies and some were or are being applied in clinical trials of NK cell infusions (11, 12, 35). Important for broader availability of these therapies are commercial sources of the necessary equipment and reagents and further development of automated systems for production of GMP-compliant clinical-grade NK cells. A pioneer in this regard is the company Miltenyi Biotec. In part as participant of NATURIMMUN, this company has further developed a protocol to expand peripheral NK cells using irradiated autologous peripheral blood mononuclear cells as feeder cells. NK cell isolation and expansion were further fully automated for future clinical applications (36, 37). NK cells generated by this procedure have been evaluated in detail (Delso-Vallejo et al., submitted to this research topic).

Another possibility is the generation of therapeutic NK cells from umbilical cord blood stem cells (UCBSC), which was pioneered by the company Glycostem (38). Within NATURIMMUN, NK cells differentiated in this system were characterized in detail and the procedure improved to yield more mature NK cells (39). Furthermore, an important role of the transcription factor ZNF683/HOBIT for NK cell differentiation could be shown supporting that the factor could be used to modulate NK cell generation [(40), this research topic]. This research topic. UCBSC-derived NK cells have been evaluated in a phase I clinical trial in elderly AML patients and found to be safe (41). Furthermore, recent evidence obtained in NATURIMMUN supports that the cells possess high cytotoxicity against metastatic colorectal cancer cells (42, 43) and could be used in the therapy of solid cancers [(44), this research topic].

An important topic in the field is to harmonize the manufacturing of GMP-compliant therapeutic NK cell products, which was initiated within NATURIMMUN and has been described in a summary of the worldwide experience obtained so far with allogeneic adaptive NK cell therapies (12). It is conceivable that expanded therapeutic NK cells could be stored frozen and be shipped on demand. These NK cells could, therefore, qualify as off-the-shelf-products, and to what extent this will be possible is a relevant question for future research.

It has been shown that expanded and cytokine-activated NK cells can be functional in certain cancer types. However, evidence suggests that specific targeting and cross-linking of NK cells to cancer cells would strongly enhance their reactivity and the applicability of NK cell therapies. A paradigm in the field is currently the exciting successes of targeting of T lymphocytes to CD19 *via* genetic CAR modification (45, 46) or corresponding bispecific reagents (46). We believe that NK cells will provide important advantages to the use of T lymphocytes based on their comparable reactivity but much higher safety. We have achieved increased NK cell cytotoxicity against leukemia cells using transduction of NK cells with CAR constructs (47, 48) or by cross-linking with trispecific reagents (Kloess et al., submitted to this research topic). Furthermore, it is conceivable that procedures to achieve redirected primary human NK cells as an “off-the-shelf-immunotherapy” can be developed. For this, optimizing both the respective antigen binding and the triggering of the intracellular signaling cascade by the CAR will be desirable (49).

A possibility to target NK cells to cancer cells can be the use of monoclonal antibody therapeutics already approved for clinical application. Examples of these are the anti-CD20 antibody rituximab (50) for B cell leukemia and the anti-EGF receptor antibody cetuximab (51–53). The latter is in use for the therapy of colon carcinoma and head and neck cancer. It displays limited efficacy in colon with better activities in head and neck cancer. It is possible that synergistic activities could be gained by coapplication of NK cell infusions as these antibodies trigger ADCC *via* binding to the low-affinity Fcγ receptor present on NK cells. It could be shown within NATURIMMUN that NK cytotoxicity toward EGFR^+^ colon and cervical cancer cells was strongly enhanced by cetuximab (42, 43). This provides a rationale to strengthen NK cell immunotherapy through a combination with cetuximab for metastatic colorectal cancer patients [(44), this research topic].

## Preclinical Models for Evaluation of Human NK Cell-Based Cancer Therapies

The preclinical evaluation of NK cell-based therapies in mouse models is hampered by the inherent problem that reagents designed to trigger human immune cell would not react at all or only partially with murine NK cells. Similarly, the evaluation of human NK cell infusions in mice does not provide a human immune cell compartment necessary for full functioning. This problem can be partly circumvented by mouse models with humanized immune system (HIS) in combination with xenotransplantation models of human cancers.

In this regard, a novel method to boost the inefficient human NK cell development in mice observed after engraftment of human HSC was recently developed. Normally, the differentiation of NK cells depends on the interplay with myeloid cells, and human myeloid cells are poorly reconstituted in available HIS mice due to competition with the murine cells (54). Therefore, a new model was developed in the NATURIMMUN project using mice that lack the Flt3 receptor (55) and display reduced murine myeloid differentiation. In these mice, human dendritic cells and consequently human NK cells could be successfully boosted by human Flt3 ligand providing a novel mouse model with increased NK cell numbers [(56), this research topic]. This will be valuable for future evaluations of immunotherapies involving reagents designed for human cells as well as human NK cell infusions.

As an exemplary preclinical evaluation, we tested within NATURIMMUN the efficacy of NK cell infusions alone or in combination with the clinically approved cetuximab against human colon cancer. HIS mice were engrafted with a human colorectal carcinoma cell line and treated with cetuximab and infusions of PB-derived and UCBSC-derived NK cells. Then the tumor load and survival rate were monitored. Significant inhibition of tumor growth and improvement of survival rates were observed. These results provide a rationale for NK infusion therapies not only for leukemia but also for solid cancer treatment [(44), this research topic].

## Main Future Directions to Achieve NK Cell-Based Cancer Immunotherapies on a Broader Scale

Collectively, the basic work on NK cells, their receptors, and NK evasion mechanisms have provided evidence for the importance of the NK cell system in the control of human cancers. Clinical trials of NK infusion therapies, performed mostly in different forms of leukemia, have uniformly shown safety of infused NK cells and in certain cases exciting effects on disease-free survival (11). This together underlines the feasibility and potential efficacy of NK cell-based immunotherapies. However, based on the currently available data a number of questions and major routes should be further explored in order for NK cell therapies to become clinically used on a broader scale. Among those are improved methods for the selection of the best donor NK cells to be able to optimally exploit the antitumor alloreactivity of NK cells (12). Then the question of best activation of NK cells by cytokines such as IL-2, IL-12, IL-15, IL-18, and IL-21 needs to be settled as reviewed within this research topic (57). In addition, the best expansion time points of clinical-scale NK cells have to be evaluated regarding both safety and efficacy with the overall goal to allow multiple adaptive NK cell application to the respective patients. The optimal application of the newly developed NK cell-directed checkpoint inhibitors needs to be explored. Further additional reagents for targeting and cross-linking of NK cells to cancer cells using bi-/trispecific antibody-based reagents should be developed to extend the range of targeted cancer cells. Similarly, additional CAR constructs for wider targeting should be derived and corresponding standard “off-the-shelf-procedures” developed for genetic modification of NK cells. Of special importance for NK infusion therapies, available technologies for NK cell generation need to be fully automated and harmonized protocols developed for large-scale GMP-compliant generation of clinical-grade therapeutic NK cells that have been recently classified as advanced therapy medicinal products in Europe. They are regulated accordingly either centralized or under hospital exemption by the member states [Regulation (EC) No 1394/2007; Directive 2001/83/EC and Regulation (EC) No 726/2004]. Given the accessibility of the tumor cells the primary focus should be on leukemia as it is to be expected that progress will be more rapid in this area. But in light of the high need of new therapies for solid cancers these should also be pursued.

## Concluding Remarks

The recent years have seen significant progress in immunotherapies of cancer based on novel checkpoint inhibitors and reagents and technology to boost T and NK lymphocytes. We propose that based on the available knowledge of NK cells, these cells will be much more amenable for therapeutic purposes based on their high cytotoxicity and generally demonstrated safety. Therefore, we suggest that a concerted effort in the development of NK cell-based immunotherapies has high potential to achieve novel therapies of hitherto untreatable and relapsed forms of leukemia and potentially also solid cancers. The development of broadly applicable NK cell-based therapies should extend the currently more restricted available T cell-based therapies and could thus boost the long-standing promise of cellular cancer therapies.

## Ethics Statement

Described work at the Medical School Hannover was carried out in accordance with the recommendations of the Ethics Committee of the Medical School Hannover with written informed consent from all subjects. All subjects gave written informed consent in accordance with the Declaration of Helsinki. The protocol was approved by the Ethics Committee of the Medical School Hannover (No 2159-2014).

## Author Contributions

EH prepared the outline, the article parts were jointly written with EH focusing on the more basic immunology parts and UK on the therapeutic aspects.

## Conflict of Interest Statement

The authors declare that the work was conducted in the absence of any commercial or financial relationships that could be construed as a potential conflict of interest.
